# Dynamic antimicrobial resistant patterns of *Escherichia coli* from healthy poultry and swine over 10 years in Chongming Island, Shanghai

**DOI:** 10.1186/s40249-022-01025-4

**Published:** 2022-09-16

**Authors:** Chao Lv, Jun Shang, Wengang Zhang, Bingqing Sun, Min Li, Chaoyi Guo, Nan Zhou, Xiaokui Guo, Shixin Huang, Yongzhang Zhu

**Affiliations:** 1 Department of Animal Health and Food Safety, School of Global Health, Chinese Center for Tropical Diseases Research, Shanghai Jiao Tong University School of Medicine, Shanghai, China; 2 One Health Center, Shanghai Jiao Tong University-The University of Edinburgh, Shanghai, 200025 China; 3 Shanghai Center for Animal Disease Prevention and Control, Shanghai Institute for Veterinary Drugs and Feeds Control, Shanghai, 201103 China

**Keywords:** Antimicrobial resistance, *Escherichia coli*, Food animal, Longitudinal trend analysis, Chongming Island, Shanghai

## Abstract

**Background:**

Antimicrobial resistance (AMR) is one of the greatest threats to animal and public health. Here, we conducted a dynamic surveillance of *Escherichia coli* on Chongming Island in Shanghai during 2009–2021 to identify the characteristics and trends of Chongming’s AMR pandemic.

**Methods:**

Rectal (cloaca) swabs from four poultry and nine swine farms (Chongming Island, 2009–2021) were collected for *E. coli* strains acquisition. The micro-broth dilution method was used to test antimicrobial susceptibility of *E. coli* isolates against 10 antimicrobial classes including 15 antimicrobials. Utilizing generalized linear mixed models (GLMMs) and co-occurrence analyses, we further explored the multiple-drug-resistance (MDR) combinations and dynamic patterns of *E. coli* over 10 years in two food animals.

**Results:**

Total of 863 MDR isolates were found among 945 collected *E. coli* isolates, 337 from poultry and 608 from swine. Both isolates exhibited high resistant rates (> 70%) to tetracyclines, phenicols, sulfonamides, penicillins, and aminoglycosides (only in swine). The resistant rates of swine isolates to penicillins, aminoglycosides, tetracyclines, phenicols, and polymyxins were significantly higher than those of poultry isolates, whereas resistance to fluoroquinolones was reversed. Resistance to polymyxins decreased similarly in swine (42.4% in 2009 to 0.0% in 2021) and poultry isolates (from 16.5% to 0.0%). However, resistance to other seven antimicrobial classes (excluding carbapenems and penicillins) declined dramatically in swine isolates, particularly fluoroquinolones (from 80.5% to 14.4%), and tendencies of resistance to the seven classes showed markedly divergent patterns in poultry isolates. Using Poisson GLMMs, the AMR carriage since 2016 was significantly lower than that of 2009 (odds ratio < 1), indicating a decline in the risk of MDR emergence. Furthermore, despite the highly diverse MDR profiles, co-occurrence analysis identified two prominent MDR clusters of penicillins-phenicols-fluoroquinolones in poultry and aminoglycosides-tetracyclines-sulfonamides-phenicols in swine.

**Conclusions:**

Our study uncovered vastly distinct AMR patterns and dynamic tendencies of poultry and swine *E. coli* isolates from Chongming. Meanwhile, Chongming’s AMR status has ameliorated, as indicated by the decline in antimicrobials prevalence (particularly in swine), lower likelihood of MDR emergence and low carbapenem-, cephalosporin-, and polymyxin resistance. Importantly, this surveillance results are the vital basis for future policy development in Chongming and Shanghai.

**Graphical Abstract:**

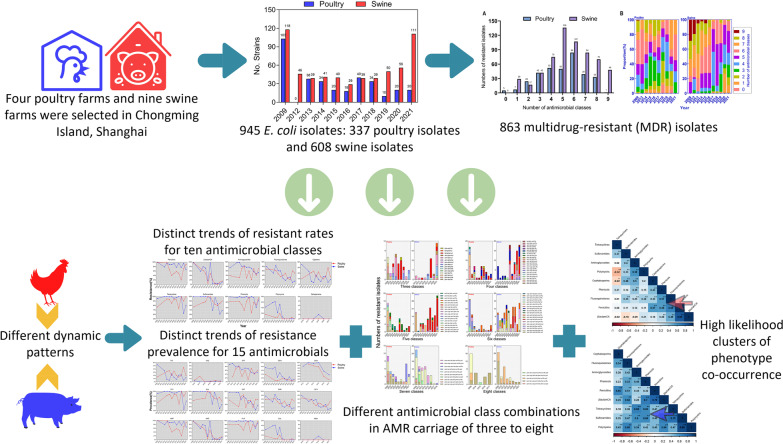

**Supplementary Information:**

The online version contains supplementary material available at 10.1186/s40249-022-01025-4.

## Background

Alexander Fleming, inventor of penicillin and pioneer of the era of antimicrobials, once warned that overuse of penicillin would cause resistant bacteria to emerge, leaving this life-saving medication ineffective [1]. Antimicrobial resistance (AMR) has, as expected, become a global concern [2–4]. In the past decades, the emergence of bacteria with multiple-drug-resistance (MDR), extensively-drug-resistance (XDR) and even pan-drug-resistance (PDR) has made the selection of antimicrobials in clinical settings more problematic. Even more concerning is the fact that a rising number of bacteria are resistant to last-resort antimicrobials, such as carbapenems, tigecycline, and colistin [5–8]. AMR is responsible for an estimated death of 700,000 people worldwide annually; this figure will have reached 10 million by 2050 [9]. Apart from the ongoing spread of AMR, the previously robust antimicrobial development pipeline has dwindled to a trickle of promising drugs [10, 11]. Dr. Tedros Adhanom Ghebreyesus, the director-general of the World Health Organization (WHO), stated at the 2019 World Health Assembly: “Together with our partners, we are intensifying the battle against antimicrobial resistance, one of the most critical health concerns of our time” [12]. Undoubtedly, China is also affected by the crisis, which is not surprising given that China is the world's largest producer and consumer of antimicrobials [13, 14].

Antimicrobials usage is primary driver of the emergence of antimicrobial-resistant bacteria; the rate of AMR increases with the use of antimicrobial drugs [15]. Approximately 73% of all antimicrobials sold worldwide are used on food animals, particularly poultry and swine produced in intensive animal production systems [16, 17]. Food animals receive low (sub-therapeutic) amounts of antimicrobials in their feed to promote growth and prevent mass infections. Despite the widespread acceptance of antimicrobials in animal husbandry in more than half of the world’s countries, this low-dose, prolonged antimicrobial use exacerbates the onset and spread of AMR [18–20]. Therefore, food animals are potential reservoirs of AMR [21]. It has been proven that China and India have the highest levels of AMR in their animal populations [22]. Importantly, antimicrobial-resistant bacteria in food animals might be transmitted directly to farm workers, and indirectly to a larger population through contaminated food, water, and soil, posing a threat to public health [23, 24].

Among the various microorganisms, *Escherichia coli* is particularly notable due to its ability to serve as a commensal in the digestive tract of humans and animals, but also to cause serious infections which may result in life-threatening or economic losses [25]. In a 2019 comprehensive study of global antimicrobial-resistant bacterial burden, *E. coli* was identified as one of the major pathogens accounting for 929,000 deaths attributable to AMR and 3.57 million deaths associated with AMR [26]. In particular, the ability of extended-spectrum β-lactamase (ESBL)-producing *E. coli* to hydrolyze third-generation cephalosporins poses a significant threat to global health [27]. *E. coli* is also the pathogen with the highest rate of clinical isolates in China [28]. Moreover, *E. coli* possesses relatively open pan-genomes, a high capacity to acquire antimicrobial resistance genes (ARGs) via horizontal gene transfer, and the ability to act as both a donor and a recipient in ARGs transmission [29]. In general, *E. coli* has been utilized as a biomarker to detect AMR in numerous medical or environmental situations, including food animal farms, hospitals, and the wildlife-livestock-human interfaces [30, 31]. In the meantime, antimicrobial-resistant *E. coli* occurred in both human and animal populations could be transmitted to a vast number of species via environmental pathways. In the context of One Health, it is also a perfect biological platform for multisector and interdisciplinary AMR disposal [29].

The majority of high-income nations have implemented AMR surveillance in animals for more than a decade; however, monitoring data on AMR are scarce in low- and middle-income countries (LMICs) [32]. Due to the high AMR burden in LMICs, robust surveillance measures are crucial, with data collection being the first vital step [26]. Shanghai, as a representative of China’s megacities, was one of the first provincial-level administrative divisions to conduct AMR surveillance on animals in 2008, with the surveillance area covering Chongming Island in 2009 [33]. Compared with other districts in Shanghai, Chongming Island is a relatively isolated region making it an ideal site to conduct long-term surveillance of AMR and evaluate the efficacy of different intervention measures. Concurrently, as one of Shanghai’s major meat, egg, and milk suppliers, Chongming Island has a direct impact on the city’s food security [34]. Furthermore, food security, human health, and environmental health related to AMR are crucial for achieving Chongming’s global ecological island goal [35, 36]. Recently, the One Health concept was adopted by the Chongming government to handle the whole island’s health issues including AMR, aimed at building an institutional basis for One Health and guaranteeing the ambitious goal of a global ecological island. In this study, we investigate the surveillance data of AMR in poultry and swine since 2009 to determine the present profiles and trends of antimicrobial-resistant *E. coli* in Chongming Island's food animals. Prior to initiating the One Health project to reduce the prevalence of AMR on Chongming Island, this study will provide a comprehensive understanding of AMR in food animals and serve as a reference for baseline data. This is, to the best of our knowledge, the first regional longitudinal investigation of AMR in food animals over a 10-year period, particularly in LMICs.

## Methods

### Sample collection and identification of *E. coli* isolates

Chongming Island, located near the estuary of the Yangtze River, was selected as the sampling sites for targeted poultry and swine farms. The sampling period began in 2009 and lasted until 2021. No isolates were collected in 2010 and 2011, and there were only swine isolates collected in 2012. At least one poultry or swine farm was chosen annually based on the following criteria: (1) Large-scale breeding farms rather than scatter-feed mode; (2) No disease outbreak in pre-selection farms; (3) The farm participated in the surveillance program voluntarily. Once a farm was selected, the sampling work is generally completed within one month in the first half of the year, and the sampling period may vary during different surveillance years. In accordance with the annual national monitoring plan for animal-derived antimicrobial resistant bacteria, at least thirty swabs of fresh feces (the sampler observed that it had just been discharged by animals) or rectal (cloaca) swabs from healthy swine and poultry were collected from each farm. For each farm, sampling must be performed within one day or within one week if the number of samples exceeds the estimated sample size. If possible, more detailed information of collected samples, e.g. the growth stages (weaned, growing) of swine and production function (layer, breeding) of poultry is obtained. All samples were collected from four poultry and nine swine farms, with poultry farm P4 being the only sampling farm since 2014 and one swine farm (S5) with a 5-year sampling period. Table 1 depicted the distribution of sampling farms over time.

**Table 1:** Location of sampling farms in Chongming Island and the number of *E. coli* isolates collected from 2009 to 2021

Year	Farm code	Species	Geolocation (longitude/latitude, E/N)	Number of isolates
2009	P1	Poultry	121.6146/31.5463	47
	P2	Poultry	121.3796/31.6928	56
	S1	Swine	121.5032/31.6646	60
	S2	Swine	121.6584/31.6287	58
Subtotal				221
2012	S3	Swine	121.3900/31.7755	46
Subtotal				46
2013	P3	Poultry	121.2269/31.7687	38
	S4	Swine	121.2341/31.7779	39
Subtotal				77
2014	P4	Poultry	121.2342/31.8189	34
	S5	Swine	121.2967/31.8462	41
Subtotal				75
2015	P4	Poultry	121.2342/31.8189	20
	S5	Swine	121.2967/31.8462	40
Subtotal				60
2016	P4	Poultry	121.2342/31.8189	18
	S5	Swine	121.2967/31.8462	29
Subtotal				47
2017	P4	Poultry	121.2342/31.8189	40
	S5	Swine	121.2967/31.8462	39
Subtotal				79
2018	P4	Poultry	121.2342/31.8189	34
	S5	Swine	121.2967/31.8462	39
Subtotal				73
2019	P4	Poultry	121.2342/31.8189	10
	S6	Swine	121.6017/31.6822	40
	S7	Swine	121.3499/31.8111	10
Subtotal				60
2020	P4	Poultry	121.2342/31.8189	20
	S6	Swine	121.6017/31.6822	18
	S7	Swine	121.3499/31.8111	20
	S8	Swine	121.5439/31.6791	18
Subtotal				76
2021	P4	Poultry	121.2342/31.8189	20
	S6	Swine	121.6017/31.6822	20
	S8	Swine	121.5439/31.6791	47
	S9	Swine	121.5016/31.7132	44
Subtotal				131
Total				945

Individual samples were collected following the routine protocols outlined below. Briefly, a sterilized cotton swab was inserted into the swine anus or poultry cloaca for 1.5–2.0 cm and rotated 2–3 times. After adding 10 ml of sterile transport medium, the feces-stained swab was stored at enclosed and homoiothermic (0–4 °C) units for shipment within 24 h. Then, plenty of transport mediums were evenly inoculated on *E. coli* coliform chromogenic media (Hopebio biotech company, Qingdao, China), and incubated at 36 °C for 18–24 h. One small, purified blue/purple spherical colony was chosen from each plate and sub-cultured for an additional 18–24 h on chromogenic media. The pure colony was then identified as *E. coli* using a microbial biochemical identification system (VITEK 2 Compact, Biomerieux, France). The confirmed isolates were stored at − 80 °C using commercial magnetic bead strain preservation tubes (Pro-Lab Microbank, Canada).

### Testing for antimicrobial susceptibility

A total of 15 antimicrobials belonging to 10 classes, namely penicillins (ampicillin), β-lactam combination agents (β-lactamCA, amoxicillin-clavulanic acid), cephems (subclass of cephalosporins III, ceftiofur and ceftazidime), penems (subclass of carbapenems, meropenem), aminoglycosides (spectinomycin and gentamicin), tetracyclines (doxycycline and tetracycline), phenicols (florfenicol), folate pathway antagonists (subclass of sulfonamides, sulfaisoxazole and sulfamethoxazole), fluoroquinolones (enrofloxacin and ofloxacin), and lipopeptides (subclass of polymyxins, colistin), were included in the antimicrobial susceptibility testing (AST). Ceftiofur, florfenicol, and enrofloxacin were only used on livestock and poultry; the use of ofloxacin and colistin on food animals has been prohibited in China since 2016; and meropenem has never been used for the treatment and prevention of disease in food animals. The minimum inhibitory concentration (MIC) values of 15 antimicrobials were determined using the microbroth dilution protocol (CLSI M100, ED32) recommended by the Clinical & Laboratory Standards Institute (CLSI) [37], and the results were interpreted using the CLSI recommend breakpoint for each antimicrobial. Three antimicrobials namely ceftazidime (2016), spectinomycin (2012) and meropenem (2016) were added to the surveillance program, and doxycycline was employed only during 2012–2017. *E. coli* ATCC25922 was selected as a quality control strain for all AST.

### Statistical analysis

The differences of phenotypic resistant rates and prevalence of resistance to antimicrobials between the hosts of poultry and swine were determined using Chi-square analysis, with a significance level of 0.05. The Chi-square tests were conducted using IBM SPSS Statistics 22 (IBM, Armonk, USA) and the graphics were created using GraphPad Prism 9 (GraphPad, San Diego, USA) and Origin 2021 (OriginLab, Northampton, USA). GraphPad Prism 9 was used to present the dynamic trends of phenotypic resistant rates and the resistance prevalence of antimicrobials.

The antibiogram length (also called “AMR carriage”) was defined as the total number of antimicrobial classes to which an isolate was phenotypically resistant [30]. Subsequently, the antibiogram length was chosen as dependent variable to test whether it differed between the two hosts and the different years by utilizing generalized linear mixed models (GLMMs), and the analysis was implemented in the “lme4” package of R 4.2.0 (Lucent Technologies, Jasmine Mountain, USA). In the meantime, the antibiogram length profiles from three to eight of poultry and swine were visualized as stacked histogram generated by GraphPad Prism 9, and two new definitions of dominant profile (the profile that emerged nearly every year and comprised the majority of isolates) and profile diversity (the number of unique profiles in a given year) were introduced. To investigate the co-occurrence of AMR phenotypes, a pairwise co-occurrence matrix of the 9 classes (excluded class of carbapenems) of antimicrobial resistance phenotypes (absence and presence) was constructed. The “polycor” package in R was adopted, and the “corrplot” package was utilized to visualize the matrix. To detect a correlation between the two phenotypes, a *P*-value of 0.05 was chosen as statistically significant.

## Results

### Sampling information of *E. coli* isolates

Over a sampling period of more than a decade (2009 to 2021, excluding 2010 and 2011) on Chongming Island, a total of 945 *E. coli* isolates were collected, comprising 337 poultry isolates and 608 swine isolates from four poultry farms (Coded P1–P4) and nine swine farms (S1–S9). Annually, the number of obtained isolates varied. The greatest number of collected isolates was 103 poultry isolates and 118 swine isolates in 2009, while the lowest number collected was 10 poultry isolates in 2019 and 29 swine isolates in 2016. Among all poultry isolates, 243 breeding-hen isolates (P1, P4) and 94 layer-hen isolates (P2 and P3) were identified. In swine, 168 isolates were collected from weaned pigs, and 440 isolates were collected from growing pigs. The distribution of farms, the sampling period, and the quantity of *E. coli* isolates collected are depicted in Table 1 and Additional file 2: Table S1.

### The status of AMR in poultry and swine *E. coli* isolates

**Fig. 1:**
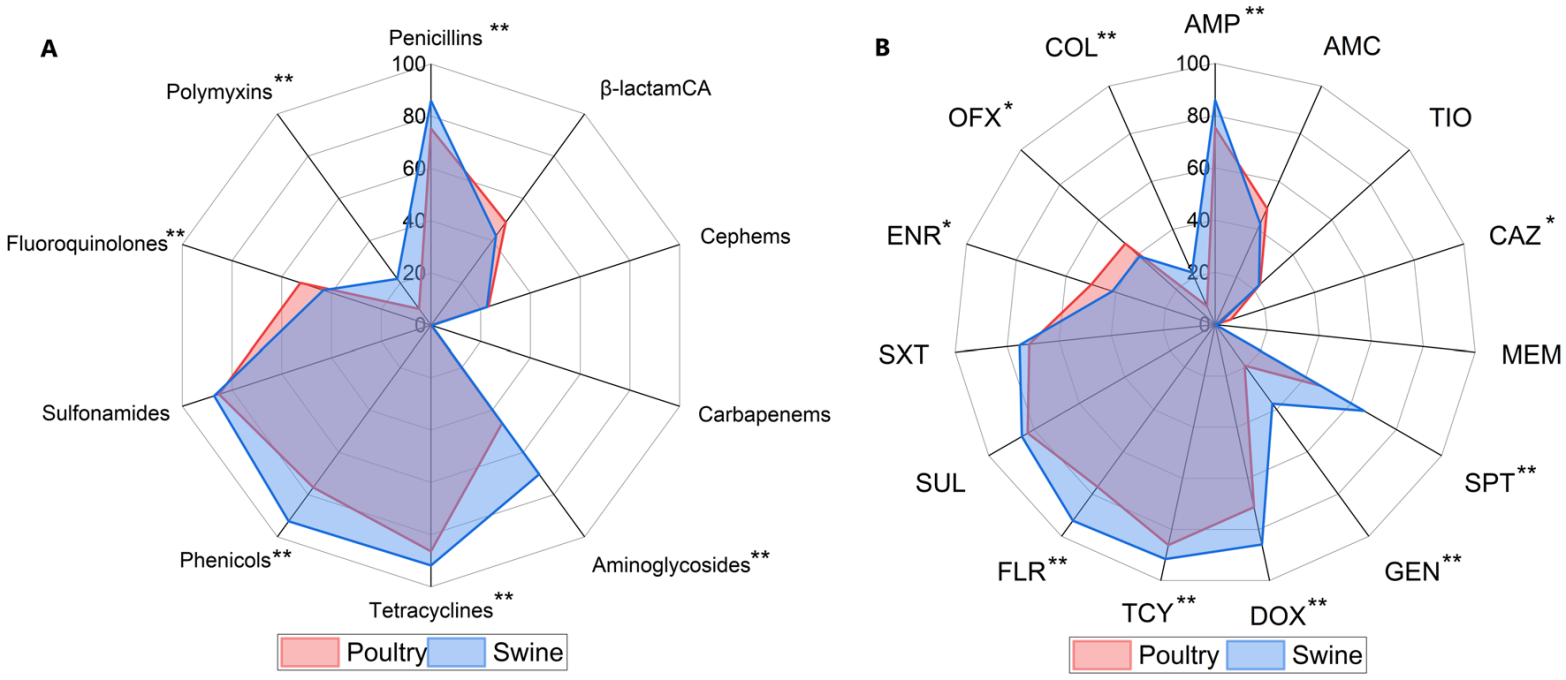
Radar charts showing percentages of *E. coli* isolates resistant to ten antimicrobial classes and fifteen antimicrobials. Asterisks indicate statistically significant variations in the resistance phenotype or prevalence of specific antimicrobials between swine and poultry, *P < 0.05, **P < 0.01. *β-lactamCA* β-lactam combination agents. *AMP* ampicillin, *AMC* amoxicillin/clavulanic acid, *TIO* ceftiofur, *CAZ* ceftazidime, *MEM* meropenem, *SPT* spectinomycin, *GEN* gentamicin, *DOX* doxycycline, *TCY* tetracycline, *FLR* florfenicol, *SUL* sulfaisoxazole, *SXT* sulfamethoxazole, *ENR* enrofloxacin, *OFX* ofloxacin, *COL* colistin

The AST results revealed that more than 70% of isolates from both poultry and swine were resistant to phenicols, tetracyclines, sulfonamides, and penicillins, with the highest resistant rate of 86.4% to tetracyclines in poultry and 92.6% to phenicols in swine. The resistant rate of aminoglycosides in swine isolates was also more than 70%. In 2019, only one swine isolate exhibited carbapenems resistance. The resistant rates of swine isolates for penicillins (*P* < 0.001), aminoglycosides (*P* < 0.001), tetracyclines (*P* = 0.008), phenicols (*P* < 0.001) and polymyxins (*P* < 0.001) were significantly higher than those of poultry isolates, whereas the resistant rates of poultry isolates for fluoroquinolones (*P* = 0.006) were significantly higher than those of swine isolates. There were no differences in resistant rates of β-lactamCA, sulfonamides and cephalosporins between the two hosts (Fig. 1A, Table 2). In addition, the resistant rates of breeding-hens, layer-hens, weaned pigs and growing pigs were shown in Additional file 1: Fig S1 and Additional file 2: Table S2.

**Table 2:** Resistant rates for various antibiotic classes and the resistance prevalence of antimicrobials in *E. coli* isolates, categorized by host

Antimicrobials classes	Poultry (%) (*n* = 337)	Swine (%) (*n* = 608)	*P*-value	Antimicrobial agents	Poultry (%) (*n* = 337)	Swine (%) (*n* = 608)	*P*-value
Penicillins	75.3	85.9	< 0.001	Ampicillin	75.3	85.9	< 0.001
β-lactam combination agents	48.7	42.3	NS	Amoxicillin/clavulanic acid	48.7	42.3	NS
Cephalosporins	23.1	22.5	NS	Ceftiofur	23.1	22.5	NS
				Ceftazidime	6.3	1.9	0.012
Carbapenems	0.0	0.3	NS	Meropenem	0.0	0. 3	NS
Aminoglycosides	46.3	70.4	< 0.001	Spectinomycin	46.6	65.7	< 0.001
				Gentamicin	19.2	37.2	< 0.001
Tetracyclines	86.4	91.8	0.008	Doxycycline	71.3	85.9	< 0.001
				Tetracycline	86.1	91.6	0.007
Phenicols	76.6	92.6	< 0.001	Florfenicol	76.6	92.6	< 0.001
Sulfonamides	85.2	87.2	NS	Sulfaisoxazole	82.8	85.4	NS
				Sulfamethoxazole	71.5	75.2	NS
Fluoroquinolones	52.5	43.3	0.006	Enrofloxacin	49.8	41.3	0.027
				Ofloxacin	46.3	39	0.029
Polymyxins	7.7	21.9	< 0.001	Colistin	7.7	21.9	< 0.001

*NS* not significant

Among all isolates from both hosts, the prevalence of resistance to ampicillin, doxycycline, tetracycline, florfenicol, sulfaisoxazole and sulfamethoxazole were more than 70%, while resistance to ceftazidime and meropenem were less than 10%. In swine isolates, the highest resistance prevalence was 92.6% for florfenicol, which was the most used phenicols class in breeding food animals. Tetracycline, another of the most extensively used antimicrobials in animal husbandry, exhibited the highest resistance prevalence among poultry isolates at 86.1%. There is no difference between the prevalence of resistance to amoxicillin/clavulanic acid, ceftiofur, meropenem, sulfaisoxazole, and sulfamethoxazole between the two hosts. While resistance to three antimicrobials (ceftazidime, enrofloxacin, and ofloxacin) was more prevalent in poultry isolates than swine isolates, the opposite was true for the remaining seven antimicrobials (Fig. 1B, Table 2). In addition, the resistance prevalence for antimicrobials of breeding-hens, layer-hens, weaned pigs and growing pigs are shown in Additional file 1: Fig S1(C, D) and Additional file 2: Table S2.

### Trends variation of resistant rates and prevalence

**Fig. 2:**
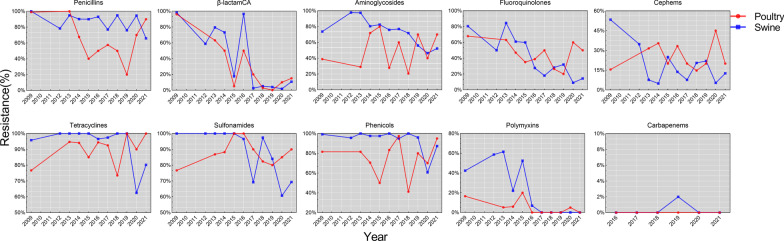
Trends in the proportion of *E. coli* isolates resistant to various antimicrobial classes from 2009 to 2021. Only one antimicrobial agent was selected for each of the classes of phenicols, polymyxins and carbapenems. *β-lactamCA* β-lactam combination agents

Three antimicrobial classes of β-lactamCA, polymyxins and carbapenems, exhibited similar trends of resistance rate between poultry and swine isolates. Since its addition to the monitoring plan in 2016, the rate of carbapenem resistance has remained almost unchanged at 0%. In contrast, downward tendencies of resistant rate were observed in the classes of β-lactamCA and polymyxins. Both poultry (from 96.1% in 2009 to 15.0% in 2021) and swine (from 98.3% in 2009 to 10.8% in 2021) isolates exhibited a considerable decline in β-lactamCA resistant rates. In addition, the resistance of poultry isolates to polymyxins decreased from 16.5% in 2009 to 0.0% in 2021, and the resistance of swine isolates to polymyxins decreased from 42.4% in 2009 to 0.0% in 2021. For the other seven classes, the resistance trends in poultry and swine isolates exhibited unique patterns. Apart from penicillins, the resistant rates of the other six classes in swine fell significantly, with the resistant rate of fluoroquinolones exhibiting the greatest decline from 80.5% in 2009 to 14.4% in 2021. Moreover, the decrease ranges for cephalosporins and aminoglycosides in swine were also larger (cephalosporins: from 53.4% in 2009 to 12.6% in 2021; aminoglycosides: from 73.7% in 2009 to 52.3% in 2021). Despite the decreasing trends of tetracyclines, phenicols and sulfonamides in swine isolates, the resistant rates of these three classes in 2021 were still more than 65.0%, and the decline of these three classes became apparent after 2018. For poultry isolates, only fluoroquinolones showed a decline trend (from 68.0% in 2009 to 50.0% in 2021), meaning that the other six classes of antimicrobials had no decline trends. In 2021, resistant rates of aminoglycosides, tetracyclines, phenicols, sulfonamides, and penicillins were all more than 70% in poultry (Fig. 2).

**Fig. 3:**
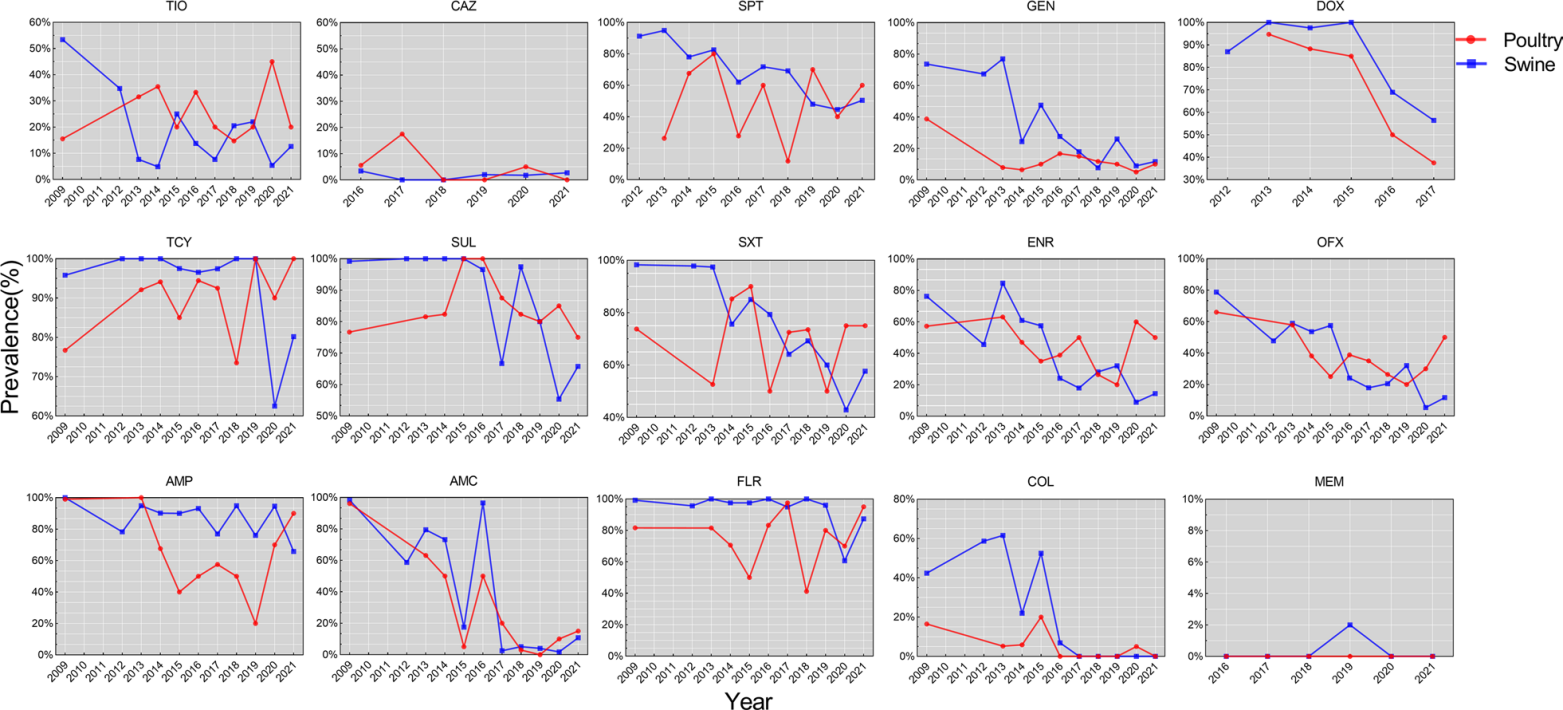
Trends in the prevalence of antimicrobial-resistant *E. coli* isolates from 2009 to 2021. *AMP* ampicillin, *AMC* amoxicillin/clavulanic acid, *TIO* ceftiofur, *CAZ* ceftazidime,* MEM* meropenem, *SPT* spectinomycin, *GEN* gentamicin, *DOX* doxycycline, *TCY* tetracycline, *FLR* florfenicol, *SUL* sulfaisoxazole, *SXT* sulfamethoxazole, *ENR* enrofloxacin, *OFX* ofloxacin, *COL* colistin

The prevalence of ceftazidime resistance was low in both poultry and swine isolates, and no discernible trends were observed. Resistance to gentamicin and doxycycline in isolates from poultry and swine exhibits a similar pattern of decreasing prevalence. However, resistance to the remaining eight antimicrobials (ampicillin, ceftiofur, spectinomycin, tetracycline, enrofloxacin, ofloxacin, sulfaisoxazole, and sulamethoxazole) varied greatly between poultry and swine isolates. Other than ampicillin, seven of the other antimicrobials showed declining trends of resistance in swine, with the most notable declines in ceftiofur (from 53.6% to 12.4%), spectinomycin (from 91.3% to 50.5%), enrofloxacin (from 76.3% to 14.4%), ofloxacin (from 78.8% to 11.7%), and sulfamethoxazole (from 98.3% to 57.7%). Comparatively, antimicrobial resistant patterns in poultry were complex, with an upward trend for tetracycline, an initially rising and then declining trend for sulfaisoxazole, a falling and rising trend for ampicillin, a slight decline trend for enrofloxacin and ofloxacin, and no discernible trends for ceftazidime, spectinomycin, or sulfamethoxazole (Fig. 3).

### MDR status and dynamics in poultry and swine isolates

**Fig. 4:**
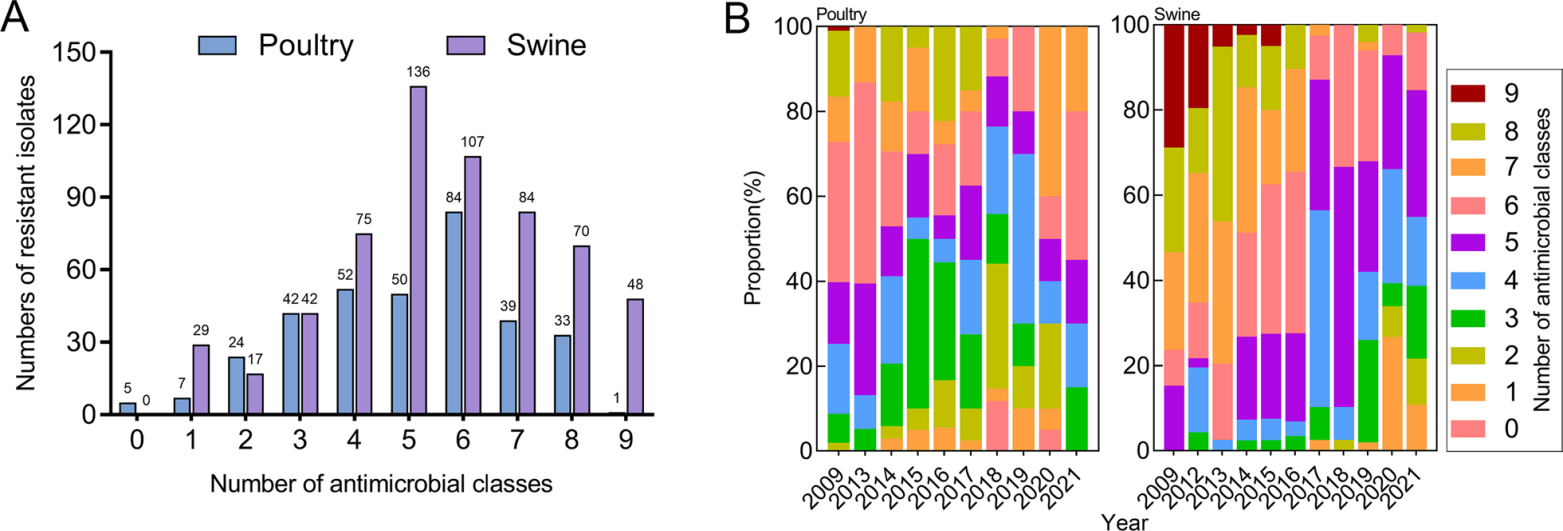
Number of antimicrobial classes (**A**) and percentage of antimicrobial classes annually (**B**)

Of the 945 isolates from poultry and swine tested, only five (four in 2018 and one in 2020) were susceptible to all 15 antimicrobials. None of the isolates were resistant to all antimicrobial classes. There were 863 (91.3%) multidrug-resistant isolates (the isolate is resistant to three or more antimicrobial classes) [38], with most being resistant to four to eight antimicrobial classes (13.4% in 4 classes, 19.7% in 5 classes, 20.2% in 6 classes, 13.0% in 7 classes and 10.9% in 8 classes; Fig. 4A). MDR distribution patterns usually fluctuated annually. While non-MDR poultry isolates peaked in 2018, non-MDR swine isolates peaked in 2020. In poultry, the isolates collected in 2013 and 2021 were all MDR, while all swine isolates collected prior to 2017 were MDRs (Fig. 4B).

The Poisson GLMMs were used to investigate the probability of AMR carriage (also called antibiogram lengths) for the hosts and the sampling years. The antibiogram length of swine was significantly longer than that of poultry [odds ratio (*OR*) = 1.19, 95% confidence interval (*CI*): 1.12–1.27, *P* < 0.001]. Based on all isolates of poultry and swine, antibiogram lengths from 2012 to 2015 did not present significant differences from those in 2009 (*P* = 0.871 in 2012, *P* = 0.811 in 2013, *P* = 0.130 in 2014, *P* = 0.404 in 2015), while antibiogram lengths in all years after 2016 were significantly shorter than that of 2009 (*OR* < 1, *P* = 0.015 in 2016, *P* < 0.001 from 2017 to 2021, Table 3).

**Table 3:** Results of a Poisson generalized linear mixed model examining the likelihood of antibiogram length within the two hosts and different years

	No. isolates	Estimate	SE	Z score	*P*-value
Hosts					
Poultry	337	Reference			
Swine	608	0.175	0.033	5.39	< 0.001
Year					
2009	221	Reference			
2012	46	0.011	0.067	0.163	0.871
2013	77	0.013	0.055	0.239	0.811
2014	75	− 0.088	0.058	− 1.515	0.130
2015	60	− 0.052	0.062	− 0.834	0.404
2016	47	− 0.175	0.072	− 2.421	0.015
2017	79	− 0.211	0.060	− 3.530	< 0.001
2018	73	− 0.302	0.064	− 4.731	< 0.001
2019	60	− 0.265	0.067	− 3.926	< 0.001
2020	76	− 0.450	0.066	− 6.804	< 0.001
2021	131	− 0.390	0.053	− 7.381	< 0.001

**Fig. 5:**
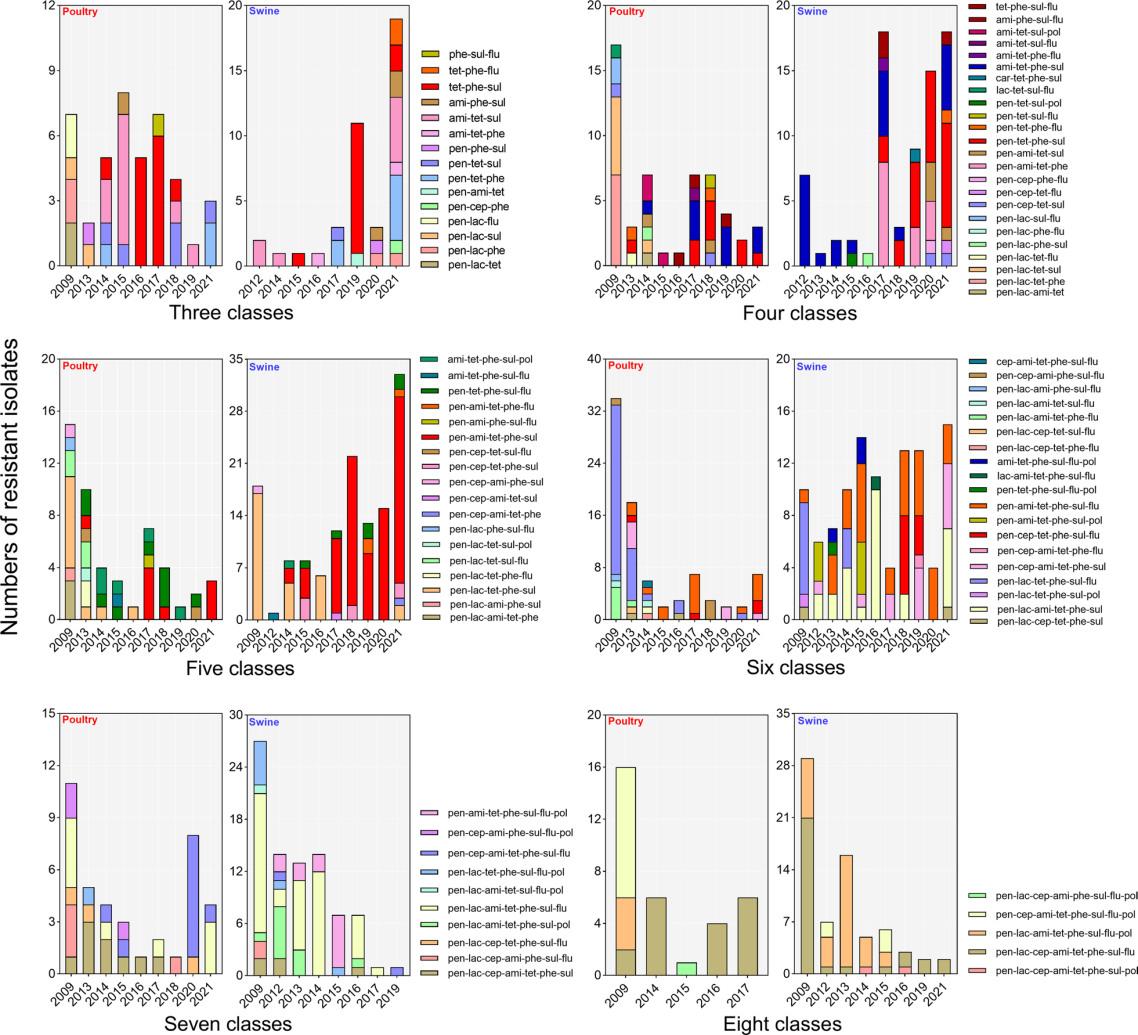
Variations and trends in antibiogram length from three and eight. *pen* β-lactam, *cep* cephalosporins, *car* carbapenems, *ami* aminoglycosides, *tet* tetracyclines, *phe* phenicols, *sul* sulfonamides, *flu* fluoroquinolones, *pol* polymyxins. Antimicrobial classes are not ranked in any particular order across all profiles

All poultry and swine *E. coli* isolates had a total of 107 unique antimicrobial resistance profiles with antibiogram lengths ranging from 1 to 9, including 37 profiles in poultry, 33 profiles in swine, and 37 profiles shared by both species (Additional file 2: Table S2). Then, we evaluated the variations and tendencies of antibiogram length profiles ranging from three to eight (Fig. 5, Additional file 2: Table S2). The two definitions of dominant profile and profile diversity described in the method section were adopted to summarize the observed phenomena. Overall, we identified the evident dominant profiles in antibiogram length of four (penicillins-tetracyclines-phenicols-sulfonamides and aminoglycosides-tetracyclines-phenicols-sulfonamides in swine), antibiogram length of five (penicillins-tetracyclines-phenicols-sulfonamides-fluoroquinolones in poultry and penicillins-aminoglycosides-tetracyclines-phenicols-sulfonamides in swine), antibiogram length of six (penicillins-β-lactamCA-aminoglycosides-tetracyclines-phenicols-sulfonamides and penicillins-aminoglycosides-tetracyclines-phenicols-sulfonamides-fluoroquinolones in swine), antibiogram length of seven (penicillins-β-lactamCA-cephalosporins-aminoglycosides-tetracyclines-phenicols-sulfonamides in poultry and penicillins-β-lactamCA-aminoglycosides-tetracyclines-phenicols-sulfonamides-fluoroquinolones in swine), as well as antibiogram length of eight (penicillins-β-lactamCA-cephalosporins-aminoglycosides-tetracyclines-phenicols-sulfonamides-fluoroquinolones in poultry and swine). None of dominant profiles were detected in antibiogram length of three (Fig. 5).

The highest diversity in poultry occurred in 2009 with 31 distinct profiles and the lowest occurred in 2019 with seven profiles, whereas the highest diversity in swine occurred in 2021 with 33 profiles and the lowest in 2018 with eight profiles (Additional file 2: Table S2). When analyzed by antibiogram lengths, the highest diversity in poultry mainly centralized in the first few years of surveillance, for instance, the highest diversity of antibiogram length of three occurred in 2014 with 6 distinct profiles. Conversely, the greatest diversity of antibiogram length profiles in swine was primarily dispersed in 2021 (except for antibiogram lengths of seven and eight in 2009 or 2012). Furthermore, the number of isolates in each profile was not uniform; poultry and swine isolates were most abundant in one or two profiles every year (Fig. 5).

**Fig. 6:**
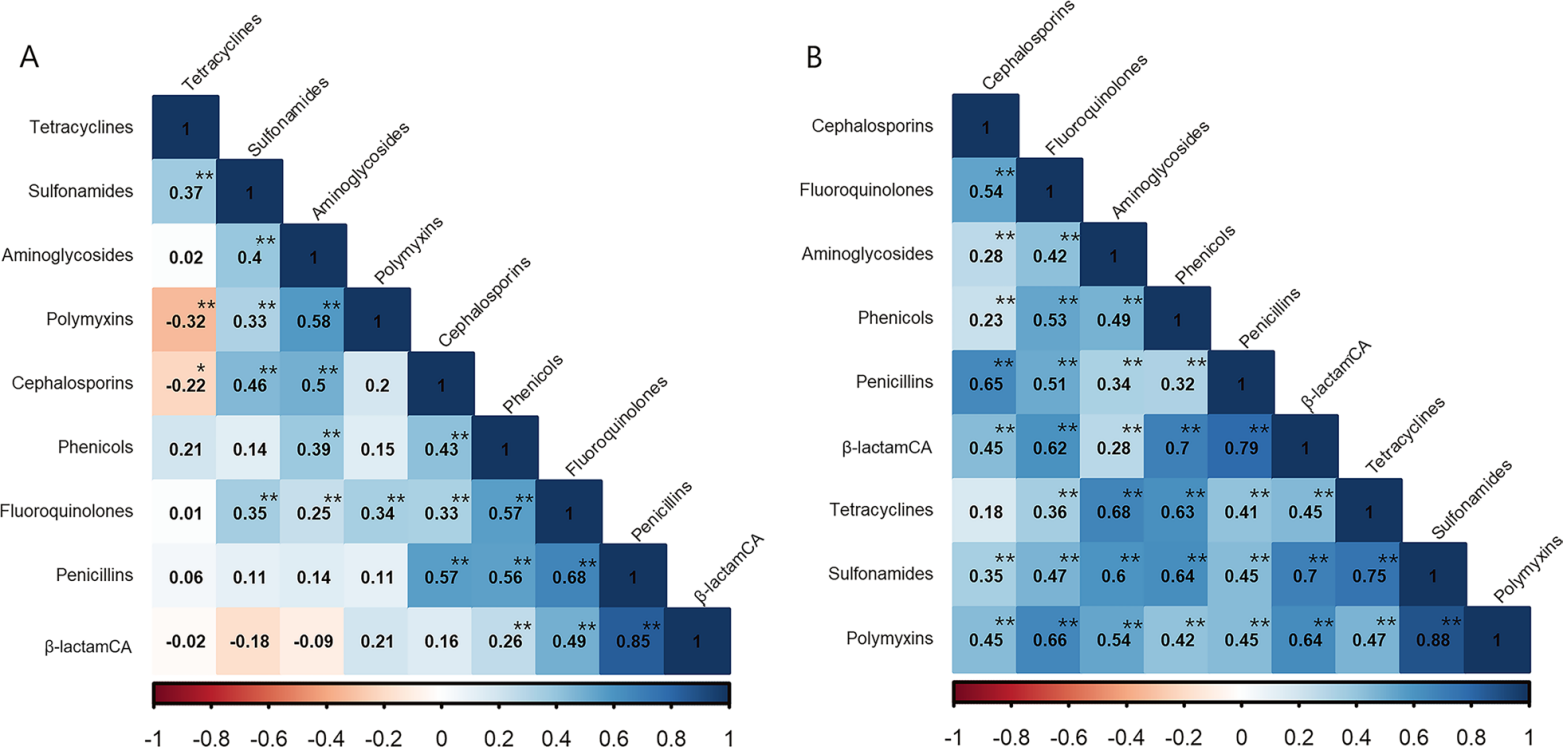
Correlations between antimicrobial resistance characteristics in *E. coli* isolates from poultry (**A**) and swine (**B**). The numbers within the boxes reflect values for the correction coefficient (*r*). The legends beneath the two heat maps indicate whether the link between resistant phenotypes is positive (closer to 1; darker blue) or negative (less than 1; lighter blue) (closer to − 1, dark red). *P < 0.05, **P < 0.01. *β-lactamCA* β-lactam combination agents

Co-occurrence analysis revealed two obvious clusters for penicillins-phenicols-fluoroquinolones in poultry and aminoglycosides-tetracyclines-sulfonamides-phenicols in swine (*P* < 0.05, correlation coefficient > 0.5). The correction coefficient of three pairs of resistant phenotypes, penicillins-cephalosporins, aminoglycosides-polymyxins and penicillins-β-lactamCA, had correction coefficients of more than 0.5 in poultry, indicating a greater likelihood of co-occurrence. Two pairs of phenotypes (tetracyclines-polymyxins and cephalosporins-tetracyclines) exhibited a negative connection; nevertheless, the correlation is weak (*r* < 0.5, Fig. 6A). Furthermore, swine isolates had a higher number of co-occurrence pairs (13 pairs) with correlation coefficients above 0.5, of which the highest pair being sulfamides-polymyxins (0.88, *P* < 0.01). It was also interesting to discover that fluoroquinolones had a higher likelihood of co-occurrence with polymyxins (0.66, *P* < 0.01), while polymyxin resistance was not the predominant resistance trait in swine (Fig. 6B).

## Discussion

In this study, a systematic surveillance of antimicrobial-resistant *E. coli* isolates in Chongming Island's food animals was conducted. This was a longitudinal study with a sample period of more than ten years, as opposed to the cross-sectional studies previously undertaken on food animal farms in various regions of China. We believe, this work complements shorter-term dynamic surveillance studies.

The prevalence of antimicrobial-resistant *E. coli* isolates in Chongming Island's food animals is alarming. Both poultry and swine *E. coli* isolates are highly resistant to tetracyclines, phenicols, sulfamates, penicillins, and aminoglycosides. Given that the aforementioned antimicrobial classes were utilized extensively in the breeding of food animals, the high resistance among them is not surprising [16, 39, 40]. Antimicrobial resistant rates (especially resistance to penicillins, aminoglycosides, tetracyclines, and phenicols) were higher in swine isolates than in poultry isolates result from significantly higher antimicrobials consumption in swine than in poultry in China [41] Furthermore, the longer feeding time in swine than in poultry may also contribute to the emergence of the high resistance phenotypes.

Since 2013, the Chinese government has issued a series of policies to regulate, restrict, and prohibit antimicrobial use in food animal breeding [33, 42, 43]. Although we do not know the variation of antimicrobial use in food animals, our surveillance results strongly support the efficacy of these measures, as AMR showed declining trends in six of nine antimicrobial classes and twelve of fifteen antimicrobials in swine isolates. In fact, there is insufficient data to explain the fluctuation of AMR in poultry isolates. In addition, the reduction in antibiogram length or AMR carriage is a further advantage of implementing these policies. Antibiogram lengths of all isolates from 2016 to 2021 were considerably shorter than those from 2009 (*OR* < 1, *P* = 0.015 in 2016, *P* < 0.001 from 2017 to 2021).

In 2015 and 2016, the Chinese government prohibited the use of ofloxacin as an antibacterial agent in food animals and the use of colistin as an animal growth-promoting feed ingredient [44, 45]. The rate of resistance to polymyxins and fluoroquinolones subsequently declined in poultry and swine isolates as a result of decreasing consumption of colistin and ofloxacin [46]. Interestingly, the prevalence of resistance to colistin has decreased to 0% in 2017 (0% in 2016 for poultry), but the incidence of resistance to ofloxacin is still over 10% (an upward trend in poultry from 2019). The discrepant mechanisms of drug resistance emergence and transmission, the difficulties of regulating farm rather than feed enterprises, and enrofloxacin’s synergistic effect in AMR (*r* = 0.88 in poultry and *r* = 0.90 in swine; data not shown) all contribute to the higher prevalence of resistant to ofloxacin.

In 2017, a list of antimicrobial-resistant bacteria that pose the greatest threat to human health was issued by WHO, including the carbapenems-resistant *Enterobacteriaceae* (CRE) [47]. Nowadays, CRE represents a worsening global threat that ignores national borders with its rapid spread [48]. In China, the class of carbapenems are not used in animal breeding, and this may be the primary reason why carbapenems-resistant *E. coli* isolates are uncommon in poultry and swine. Here, the source of the single carbapenem-resistant isolate in swine is unclear based on the available data. However, we assume that the emergence of the carbapenem-resistant isolate might be sporadic because it was not discovered during the subsequent 2 years.

Whole-genome sequence undertaken on 31 Swiss farms revealed that clonal transmission from animal to animal or via contaminated stable surfaces, rather than the introduction of new strains, was the predominant mode of transmission [49]. Several dominant profiles (which appear in multiple years and have a preponderance of isolates number) were identified in our study, and we infer that they may persist in the farm in the absence of all-in and all-out management and good hygiene. In addition, some resistant phenotypes in poultry or swine may be synergistic [30]. As demonstrated by this study, penicillins-phenicols-fluoroquinolones in poultry and aminoglycosides-tetracyclines-sulfonamides-phenicols in swine are evident co-occurrence phenotype clusters (*r* > 0.5, *P* < 0.01), indicating potential circulating conjugative MDR plasmids within the *E. coli* population of the two hosts, particularly the cluster of aminoglycosides-tetracyclines-sulfonamides. This is because ARGs conferring resistance phenotypes to tetracyclines and sulfonamides are typically attributed to mobile genetic elements [50, 51].

One of the five objectives of the Global Action Plan on Antimicrobial Resistance issued by WHO, Food and Agriculture Organization, and World Organization for Animal Health, is to strengthen the knowledge and evidence basis through surveillance [52]. AMR surveillance in animals is inadequately documented in LMICs, particularly data on continuous, systematic longitudinal surveillance [32]. Local prevalence surveys are imperfect substitutes for surveillance networks and may not reflect the dynamics of AMR within farms, even in the regions, or national level. Several investigations on the dynamics of extended-spectrum cephalosporin-resistant *E. coli* (ESC-R-Ec) in European pig farms concluded that longitudinal surveillance is necessary since the presence of ESC-R-Ec fluctuates throughout a pig’s lifetime [53, 54]. Meanwhile, as the world's largest producer of poultry and swine as well as the greatest producer and consumer of antimicrobials, understanding the current status of AMR in food animals in China is crucial for worldwide AMR surveillance [16, 32]. According to Daniel et al. systematic review, eastern China and India will benefit the most from future surveillance initiatives [55]. Consequently, this 10-year longitudinal surveillance of AMR in food animals on Chongming Island is part of China's animal AMR surveillance network and a supplement to the absence of animal AMR surveillance in LMICs.

Chongming Island is a suitable site for AMR surveillance due to its geographical location and thriving animal husbandry. In the process of constructing the global ecological island, the potential threat to environmental health, food security, and public health posed by AMR generated from food animals must be addressed. Nowadays, One Health is regarded as the most effective strategy to combat health risks, such as zoonosis, AMR, food security, and so on [56–58]. Chongming Island will be regarded as the ideal region for implementing One Health to combat AMR. In addition, our research demonstrates that swine can serve as an animal model for evaluating anti-AMR strategies. Moreover, there are two major limitations to our research. The lack of critical data from animal farms, such as the number of animals present, antimicrobials used, and disease reports, as well as the low number of animal samples, is the primary limitation of our study. Secondly, the study lacks genomic sequence data. Combining genomic ARGs and mobile genetic elements with phenotypic data will offer more convincing information on how to combat AMR.

## Conclusions

Our study focused on Chongming Island’s antimicrobial-resistant *E. coli* isolates from food animals during 2009–2021 revealed an alarming scenario with > 90% MDR phenotypes and high resistance to phenicols, tetracycline, sulfonamides, penicillins, aminoglycosides, and fluoroquinolones. The AMR trend patterns of poultry and swine isolates are distinct: the resistance phenotypes to the majority of antimicrobial classes and the prevalence of resistance to the majority of antimicrobials decreased in swine isolates, while poultry isolates exhibited less pronounced downward trends. AMR carriage in swine isolates was much higher than in poultry, and distinct MDR clusters were discovered, as determined by GLMMs and co-occurrence analyses. Considering food animals are the primary source of animal protein for humans, the One Health approach should be implemented to coordinate the public health and veterinary sectors in order to combat the AMR threat. In addition, this surveillance study on food animals is an important contribution to the food animal surveillance network in LMICs.

## Supplementary Information

**Additional file 1: Figure S1.** Radar charts showing percentages of *E. coli* isolates of breeding-hens, layer-hens, growing pigs, and weaned-pigs origin resistant to ten antimicrobial classes (A, B) and fifteen antimicrobials (C, D). Asterisks indicate statistically significant variations in the resistance phenotype or prevalence of specific antimicrobials between swine and poultry, * *P* < 0.05, ** *P* < 0.01. *β-lactamCA* β-lactam combination agents, *AMP* ampicillin, *AMC* amoxicillin/clavulanic acid, *TIO* ceftiofur, *CAZ* ceftazidime, *MEM* meropenem, *SPT* spectinomycin, *GEN* gentamicin, *DOX* doxycycline, *TCY* tetracycline, *FLR* florfenicol, *SUL* sulfaisoxazole, *SXT* sulfamethoxazole, *ENR* enrofloxacin, *OFX* ofloxacin, *COL* colistin.

**Additional file 2: Table S1.** The quantity of *E. coli* isolates from the involved farm in Chongming Island from 2009 to 2021. **Table S2.** Resistant rates for various antimicrobial classes and the resistance prevalence of antimicrobials in *E. coli* isolates, categorized by host of different production functions. **Table S3.** Profile composition and number of *E. coli* isolates from human and animal in Chongming Island, Shanghai.

## Data Availability

Not applicable.
