# Supplementary figures and images for: Dynamic antimicrobial resistant patterns of Escherichia coli from healthy poultry and swine over 10 years in Chongming Island, Shanghai
Source: Infect Dis Poverty. 2022 Sep 16;11:98. doi: 10.1186/s40249-022-01025-4 (PMC9482194; doi:10.1186/s40249-022-01025-4)

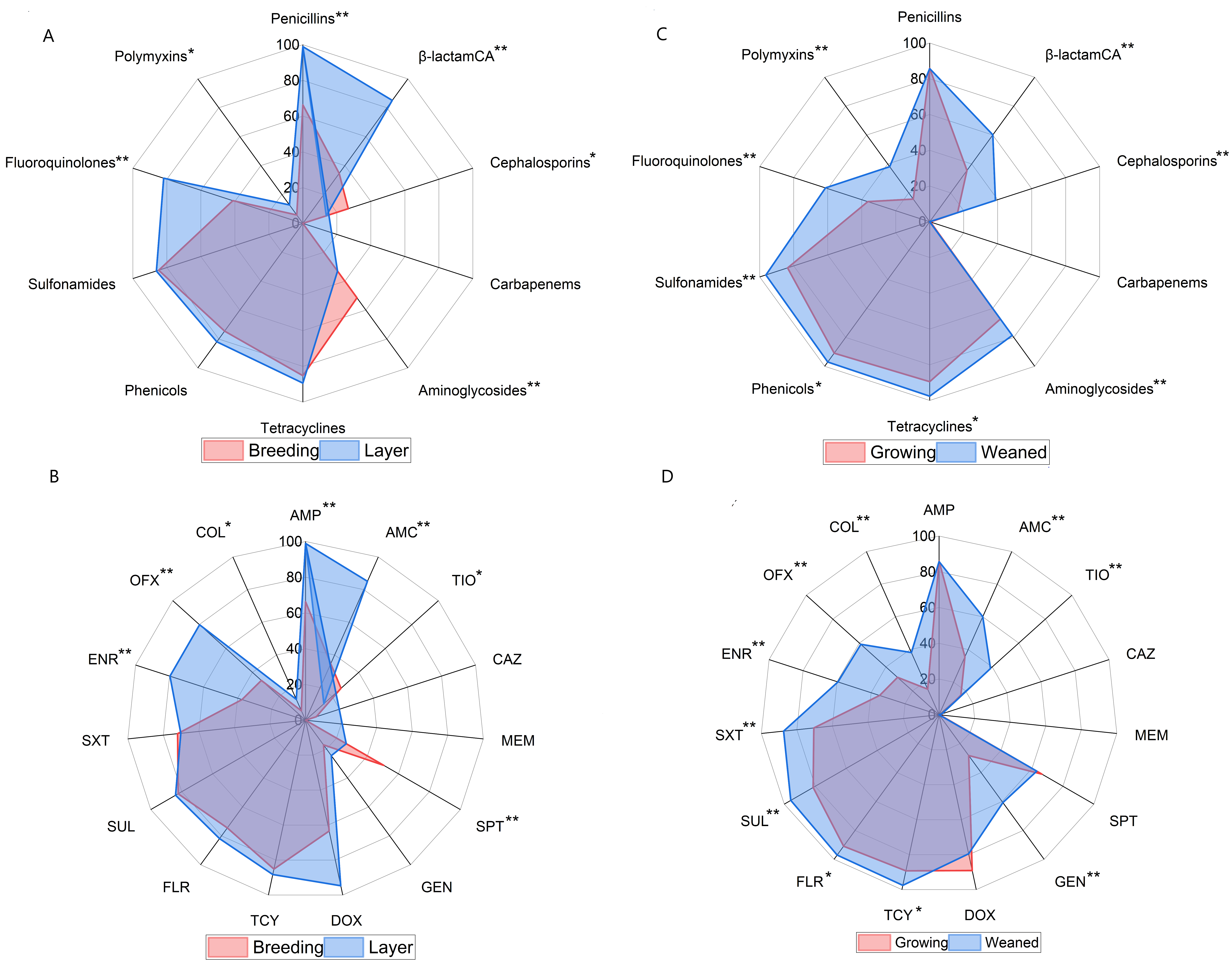

Supplement: Supplementary file 1 — Additional file 1: Figure S1. Radar charts showing percentages of E. coli isolates of breeding-hens, layer-hens, growing pigs, and weaned-pigs origin resistant to ten antimicrobial classes (A, B) and fifteen antimicrobials (C, D). Asterisks indicate statistically significant variations in the resistance phenotype or prevalence of specific antimicrobials between swine and poultry, * P < 0.05, ** P < 0.01. β-lactamCA β-lactam combination agents, AMP ampicillin, AMC amoxicillin/clavulanic acid, TIO ceftiofur, CAZ ceftazidime, MEM meropenem, SPT spectinomycin, GEN gentamicin, DOX doxycycline, TCY tetracycline, FLR florfenicol, SUL sulfaisoxazole, SXT sulfamethoxazole, ENR enrofloxacin, OFX ofloxacin, COL colistin. [file 40249_2022_1025_MOESM1_ESM.png]
